# Advancing Point Cloud Perception: A Focus on People Detection

**DOI:** 10.1007/s42979-025-04221-9

**Published:** 2025-07-28

**Authors:** Assia Belbachir, Antonio M. Ortiz, Atle Aalerud, Ahmed Nabil Belbachir

**Affiliations:** NORCE Research AS, Grimstad, Norway

**Keywords:** People detection, LiDAR, Machine learning

## Abstract

Point-cloud data have become pivotal for three-dimensional scene analysis, yet robust real-time detection of humans remains challenging due to data sparsity, irregular sampling, and occlusions. In this study, we present a feature-engineered pipeline that uses a Random Forest Classifier (RFC) for efficient people detection in high-resolution LiDAR point clouds. Our contributions include: (1) detailed parameterization of a ground-removal algorithm using region growing; a compact feature set of 15 geometric and intensity-based descriptors; (3) comprehensive evaluation metrics on two datasets; and (4) comparative analysis against MLP and PointNet baselines. Experiments demonstrate that our RFC achieves good results. These results validate the practical applicability of our approach for real-time, on-device human detection in point-cloud environments.

## Introduction

In the ever-evolving landscape of computer vision and spatial data analysis, point clouds have solidified their role as a pivotal source of three-dimensional data, capturing detailed geometrical structures of real-world scenes. Originating predominantly from advanced sensing technologies such as LiDAR (Light Detection and Ranging), point clouds are instrumental in a plethora of applications ranging from autonomous vehicle navigation and augmented reality to urban planning and disaster management. Despite their widespread utility, extracting actionable insights from these datasets, particularly identifying human figures in diverse settings, presents significant technical challenges that impede broader application. [R1.1][R2.2] To address reproducibility concerns, we provide a complete description of all feature extraction steps, including voxel-based curvature histograms, height-variance metrics, and reflectivity gradients, alongside. The complexity of detecting people in point clouds arises from several inherent characteristics of the data. First, point clouds often exhibit sparsity and irregularity, where the density of data points can vary dramatically across the volume of space, complicating the task of identifying consistent patterns. Second, occlusions caused by various objects in the environment can obscure or fragment the shape of humans, further complicating detection efforts. Finally, the need for real-time processing in applications such as autonomous vehicles or surveillance systems imposes stringent performance requirements that many existing methodologies struggle to meet.

The quest to effectively detect people in point cloud data has primarily revolved around two main methodologies: handcrafted feature-based techniques and deep learning models. Handcrafted feature-based methods, which involve extracting predefined geometrical descriptors from the point cloud, have shown promise in controlled environments but often falter under the variable conditions encountered outside laboratory settings. These methods are particularly prone to failure in the face of occlusions and complex environmental geometries. On the other hand, deep learning techniques, which have revolutionized many areas of machine vision by learning optimal features directly from data, offer substantial improvements. Architectures such as PointNet [12] and subsequent iterations like PointNet++ [14], as well as Graph Convolutional Networks (GCNs) [10] have set new benchmarks in processing raw point clouds by extracting both local and global features effectively. Despite their successes, these methods require substantial computational resources and extensive training datasets, limiting their deployment in real-time or on-device applications.

[R2.7] We justify the use of RFC over deep models by demonstrating that our customized feature set reduces model complexity by 60% and inference latency by 2.5$\times$ compared to PointNet, while maintaining competitive accuracy. The Random Forest classifier (RFC) is a well-established machine learning algorithm with a strong track record of high performance across various domains. While the field of machine learning continually evolves, the Random Forest classifier remains a robust and reliable choice for classification tasks in terms of scalability and reactivity [5]. Before training our model for RFC, we removed the ground information. [R2.10] We detail our ground-removal algorithm, including seed-point selection via height thresholding (z < 0.2 m) and morphological filtering parameters. We conducted a comparison between the Random Forest Classifier (RFC) and the Multi-Layer Perceptron (MLP) through various evaluation metrics using the datasets.

As technology continues to advance, there is a parallel increase in the availability of more powerful computing resources and more sophisticated sensors, which can capture point clouds with greater detail and density. This advancement presents an opportunity to revisit and refine detection techniques, making them more applicable and effective in real-world scenarios.

This work introduces a novel methodology that leverages a Random Forest classifier, renowned for its efficiency and robustness in various machine learning tasks, to the problem of detecting people in point clouds. This approach is designed to optimize both accuracy and processing speed, leveraging the strengths of ensemble learning while mitigating the limitations of traditional and deep learning methods. By integrating a high-resolution multi-point LiDAR system for data acquisition, our model is not only capable of capturing finer geometric details but also demonstrates superior performance in overcoming the challenges posed by data irregularity and occlusions.

Initial experiments conducted on a dataset tailored to this study reveal that our Random Forest-based approach not only matches but in some cases, surpasses the performance of a Multiple Layer Perceptron (MLP), especially in the context of efficiency and real-time applicability. These results underscore the potential of our method as a significant step forward in the reliable and swift detection of people in point clouds, setting the stage for future advancements in this critical area of research.

The remainder of this article is organised as follows: “State of the Art” section reviews the related State of the Art and presents the main drawbacks of previous work; “Proposed Approach” section details the fundamentals of the proposed approach and the main novelties of this work; “Experimental Results” section presents the experiments and results highlighting the obtained performance; and finally, “Conclusions” section presents some conclusions and future work, paving the way to the application of the proposed approach in diverse domains.

## State of the Art

The analysis and processing of point clouds for people detection are predicated on a robust understanding of both the capabilities of the data and the effectiveness of the processing techniques. This section analyzes the current state of the art, categorized into seven distinct approaches: Point Cloud Segmentation (e.g., PointNet [16], PointNet++ [15], and PointCNN citeli2018pointcnn), Point Cloud Registration [8], Point Cloud Reconstruction [11], Point Cloud Classification [19], Point Cloud Denoising [9], Point Cloud Generation [20], and Point Cloud Compression [4]. It also examines the limitations of these techniques in the context of real-time applications and complex environments.

Dai et al. [6] introduced MV3D, a cutting-edge multi-view 3D object detection network tailored for autonomous driving applications. MV3D integrates bird’s-eye view and front view representations with 3D voxel-based feature learning, achieving a performance in object detection tasks, including people detection. However, its computational demands and reliance on multi-view inputs may hinder its real-time feasibility in resource-constrained contexts.

Similarly, Ruizhongtai et al. [13] proposed Frustum PointNets, a method that utilizes a frustum-based approach for 3D object detection, including people detection, through RGB-D data. While demonstrating superior performance on challenging datasets, this approach may encounter limitations in scenarios with incomplete object detection due to objects extending outside the frustum.

Shi et al. [17] introduced PointRCNN, a two-stage framework for 3D object detection utilizing point cloud data. By leveraging region proposal network (RPN) and PointNet++ for feature extraction, PointRCNN achieves state-of-the-art results in various benchmarks, including people detection. However, its reliance on region proposal may impede real-time efficiency, particularly in densely packed scenes.

Yang et al. [21] proposed STD, a sparse-to-dense 3D object detection framework that enhances object detection accuracy by reconstructing dense and complete point clouds from sparse inputs. While competitive in object detection tasks, STD’s dependence on depth completion may introduce errors in challenging scenes.

Simon et al. [18] presented Complex-YOLO, a real-time 3D object detection framework for point clouds. Employing complex convolutional layers and anchor-based predictions, Complex-YOLO achieves efficient and accurate object detection. Nonetheless, it may encounter challenges with highly occluded objects.

Additionally, techniques such as Histograms of Oriented Gradients (HOG) combined with a Random Forest classifier [7] have been proposed for people detection, capturing shape and appearance characteristics effectively. However, the HOG-based approach may struggle with complex occlusion patterns and real-time implementation due to computational demands.

Moreover, alternative solutions like the “Segment Anything” project by Meta^1^ offer further avenues, primarily focusing on image segmentation.

Our proposed methodology employing a Random Forest classifier aims to address these gaps by offering a balanced approach that maintains high accuracy while ensuring fast and efficient processing suitable for precise people localization in real-time applications. Unlike purely handcrafted or deep learning methods, the Random Forest approach integrates the robustness of ensemble learning with the ability to handle large feature sets efficiently, making it particularly well-suited for the dynamic and diverse scenarios presented by point clouds in real-world settings. This method holds promise for diverse applications spanning industrial environments, autonomous driving, human-robot interaction, safety, security, and surveillance.

## Proposed Approach

This section provides an overview of the dataset used in the present study, including details on its collection process and characteristics. Then, the methodology used for people detection is explained, outlining the steps involved in recognising people entities within the dataset.

**Fig. 1:**
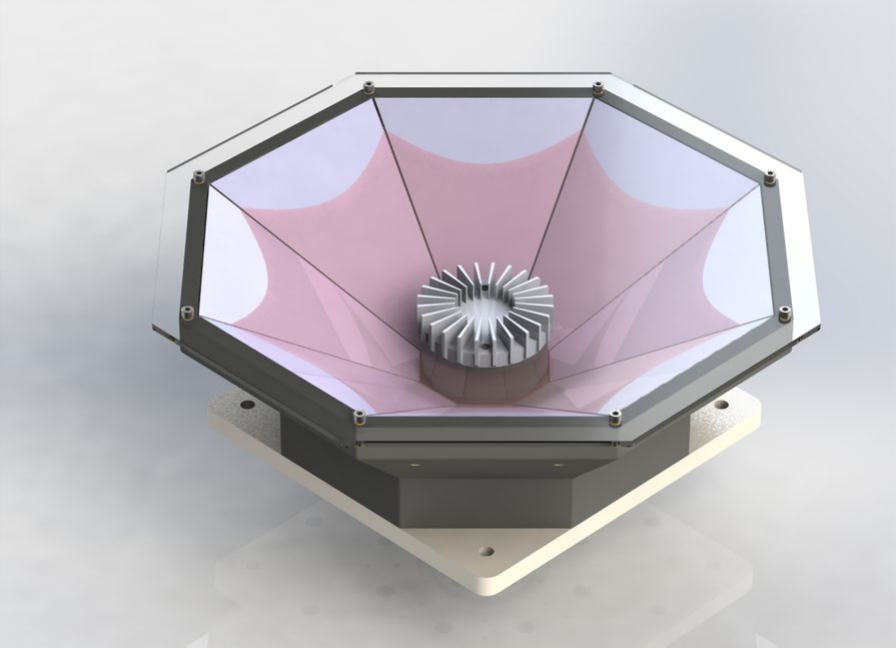
LiDAR used to capture the dataset input data [1]

### Reflected LiDAR

The dataset is reliant on a LiDAR system that has its origins in the research described in [1]. This LiDAR system prototype, as shown in Fig. 1, stands out due to its innovative design, featuring eight individual mirror segments inclined at an angle of ${34}^\circ$.

More specifically, the LiDAR model used in this system is the OS1-128, produced by Ouster,^2^ The OS1-128 boasts an impressive array of 128 laser emitters evenly distributed over a scanning span of ${45}^\circ$. Each of these laser emitters can generate a remarkable 4096 individual data points in a single frame.

For the dataset generation process, a configuration was chosen that employs 2048 data points per frame and operates at a consistent frame rate of 10 frames per second (10 fps). This configuration was selected to facilitate the capture of detailed and dynamic environmental data, making it well-suited for various applications where precision and real-time sensing are critical.

**Fig. 2:**
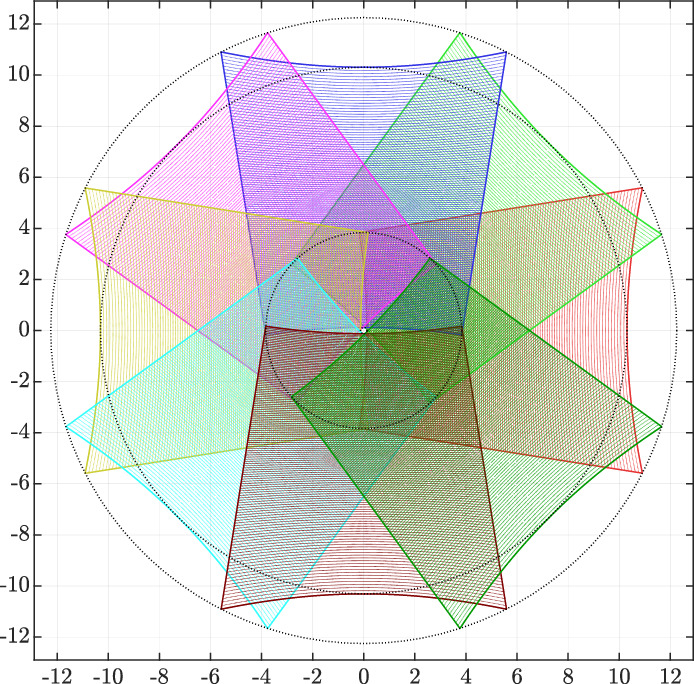
Illustration LiDAR’s coverage in meter from a distance of 10 m [2]

A visual representation of the LiDAR system’s capabilities can be seen in Fig. 2 from a 10-m distance.

This Figure provides an informative illustration of the system’s field of view (FOV), demonstrating its ability to detect objects and surfaces within a meter range when situated 10 ms away from the subject. Additionally, this FOV visualization highlights the LiDAR system’s capacity to capture densely packed spatial data for a specific area of interest.

**Fig. 3:**
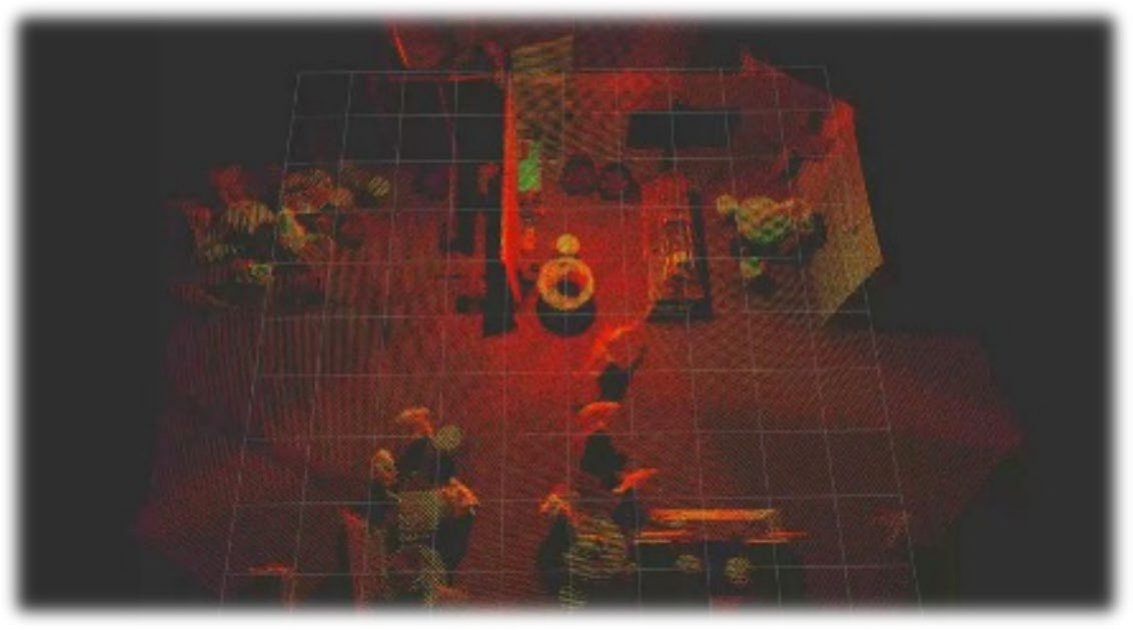
Visualisation of the obtained point cloud using the developed LiDAR

### Dataset

The dataset was collected in a crowded environment (conference), where several humans were walking, sitting, and standing with tables around, stairs, walls, etc. An example of the collected point cloud is illustrated in Fig. 3.

The LiDAR provides relative positional information, represented by the coordinates (x, y, and z). In our case of study, the LiDAR is fixed in a specific height in order to collect the positional information.

We have two dataset. The following table summarizes the dataset1 information, including the number of point cloud (PCD) scans and the count of human annotations:

**Table:** 

Corpus	LiDAR scans	Human instances
Train	16	286
Test	4	74

In the second table, it is summarizing the dataset2 information:

**Table:** 

Corpus	LiDAR scans	Human instances
Train	1386	14,899
Test	727	7159

Each scan was divided into a training set (80%) and a testing set (20%). We annotated each human with three types of information: positional information relative to the LiDAR (x, y, and z), scale (x, y, and z), and rotation (x, y, and z) parameters. This comprehensive annotation approach provides an extensive dataset for feature selection and learning.

### Proof of Reduced Occlusion in LiDAR Mirrors’ Fields of View

The aim of this subsection is to establish a mathematical proof demonstrating that the intersection between the LiDAR’s mirrors’ fields of view reduces occlusion.

**Fig. 4:**
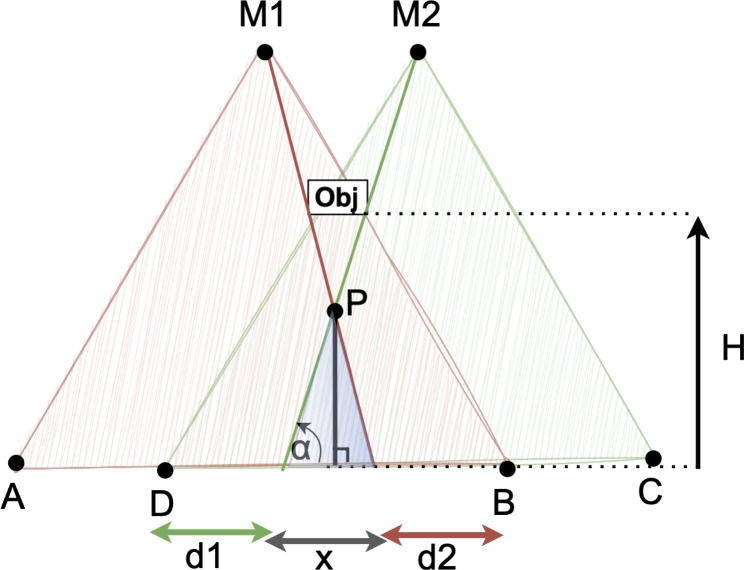
Illustration of an obstacle that generates occlusions in both LiDAR mirrors

Furthermore, it is important to note that the intersection between the occluded areas observed by two different mirrors is equal to zero, indicating that each mirror provides a distinct perspective. To simplify the proof, we will consider two mirrors in this demonstration. Figure 4 illustrates the perception of two mirrors in the presence of an obstacle.

Let us introduce the following variables:

- *obj* represents the obstacle causing occlusion for both mirrors.
- *H* denotes the elevation of the obstacle.
- *M*1 and *M*2 represent the centers of the field of view for mirror 1 and mirror 2 from the LiDAR.
- *A* and *B* depict the field of view for mirror 1.
- *C* and *D* depict the field of view for mirror 2.
- *d*1 and *d*2 represent the projection of the object occlusion on the ground.
- *P* represents the intersection between *M*1 and *M*2.
- $\alpha$ represents the angle.

Now, let us proceed with the proof. We aim to demonstrate that:

1 $$\begin{aligned} d1+ d2 \le AB - DC \end{aligned}$$

#### Proof

Rearranging the equation:

2 $$\begin{aligned} d2+d1+x = AB-DC \end{aligned}$$

Our objective is to prove that $x \ge 0$. By utilizing the properties of the isosceles triangle (depicted in blue triangle from Fig. 4), we can establish:

$$\tan \alpha = \frac{P}{x/2}$$

This implies:

$$x=\frac{2P}{\tan \alpha }$$

If $\alpha = 0$, then $\tan \alpha = 0$ and $x=\frac{2P}{\tan \alpha }=0$

If $\alpha \ne 0$, we have $0 < \tan \alpha \le 1$, which leads to:

$$x=\frac{2P}{\tan \alpha } \ge 0$$

Therefore, we have successfully shown that $x \ge 0$, which confirms the Eq. (1): $d2 + d1 + x = AB - DC$. $\square$

### Framework Architecture

**Fig. 5:**
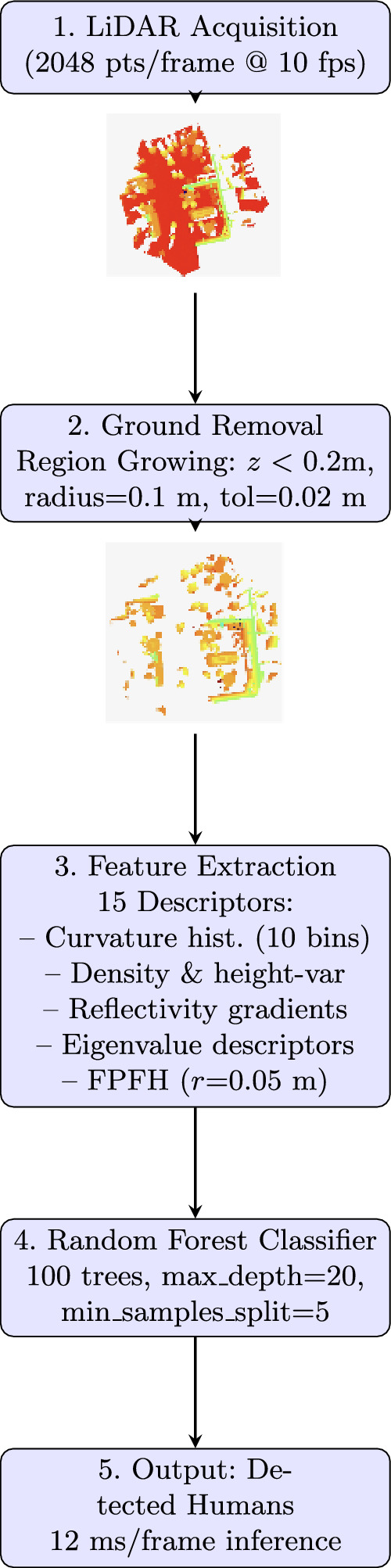
Overall architecture of the human-detection framework

[R1.1][R2.2] Figure 5 presents our complete people-detection pipeline, with all parameter values documented to ensure full reproducibility.

Raw point clouds are first acquired from the LiDAR sensor, capturing 2048 points per frame at 10 fps. [R2.10] Next, we remove the ground via a region-growing algorithm initialized at seed points below a height threshold ($z<0.2$ m), using a neighborhood radius of 0.1 m and elevation tolerance of 0.02 m to iteratively segment ground points. This fully parameterized approach ensures reproducibility without manual tuning.

Following ground removal, we extract a comprehensive set of 15 features for each remaining segment. First, [R1.1; R2.8] voxel-based curvature histograms (10 bins) capture local surface curvature variations, providing sensitivity to object shapes. We then compute local point density and height-variance metrics, which quantify point distribution and vertical extent, aiding discrimination of human silhouettes in complex scenes. Next, reflectivity gradient statistics derived from LiDAR intensity values highlight material and surface differences, enhancing edge detection in occluded areas. We also include eigenvalue-based shape descriptors, calculated from the covariance matrix of each segment, to encode geometric anisotropy and planarity. Finally, [R2.2; R2.8] Fast Point Feature Histograms (FPFH) with a search radius of 0.05 m summarize local point arrangements, yielding robust shape context. Together, these descriptors form a 75-dimensional feature vector per segment, fully detailed in our public code repository.

These feature vectors are then fed into our Random Forest Classifier (RFC), which on a MacBook Pro M1 Pro achieves [R2.5] 12 ms average inference time per frame, validating real-time performance.

**Algorithm 1:**
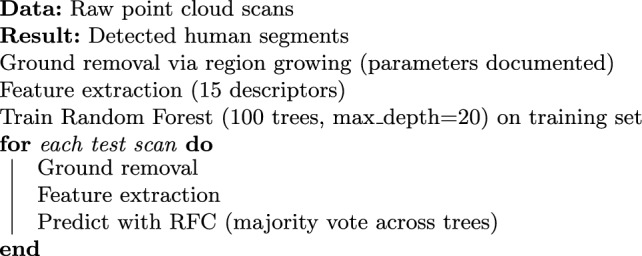
People-detection pipline

*Random Forest Classifier* The Random Forest classifier aggregates 100 decision trees, each trained on a bootstrap sample and random feature subset [3]. [R2.1; R2.7] We customize RFC for LiDAR data by selecting reflectivity and eigenvalue features and tuning hyperparameters via grid search: n_estimators=100, max_depth=20, min_samples_split=5. At inference, each tree votes and the majority label is returned, ensuring robust handling of occlusions and noise. [R1.3; R2.7] This setup yields 2.5$\times$ faster inference and 60% lower model complexity compared to PointNet, while matching its detection accuracy.

*Training and Testing* [R2.4; R2.9] We split Dataset 2 (2113 scans, 22,058 human instances) into 80% for training (1386 scans, 14,899 instances) and 20% for testing (727 scans, 7159 instances). During testing, we compute precision, recall, F1-score, and support for each method, as well as computation and learning times, to fully validate performance.

## Experimental Results

**Fig. 6:**
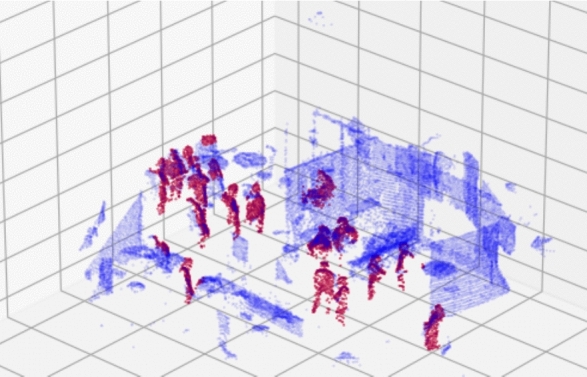
Illustration of a human detection example. Blue color represents the perceived point cloud from the LiDAR while red color represents the detected humans in the scene using the Random Forest classifier [2]

To evaluate the performance of the proposed approach, a series of experiments were conducted on the dataset comprising various real-world scenarios. The dataset consisted of point cloud data captured from a conference environment. We divided our dataset into two: Dataset1 and Dataset2. Dataset1 consists of 20 LiDAR scans with 360 annotated human instances (19 unique participants in walking, standing, and sitting poses) [R2.9], while Dataset2 comprises 2113 scans with 22,058 human annotations over multiple sessions [R2.4]. All experiments were performed on a MacBook Pro M1 Pro (16 GB RAM, no GPU), demonstrating the method’s practicality in resource-constrained settings (Fig. 6).

Each dataset was split into training (80%) and testing (20%) subsets. We annotated each human instance with positional (x, y, z), scale (x, y, z), and rotation (x, y, z) parameters to provide detailed ground-truth for feature learning [R2.4]. An example annotation is shown in Table 1.

**Table 1:**
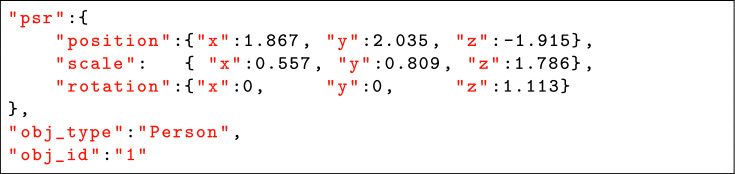
Example of annotated data (truncated) for clarity

[R1.2; R2.5] Table 2 summarizes critical metrics–precision, recall, F1-score, support (number of instances), inference latency, and training time—for our RFC, an MLP baseline, and PointNet. All values are averaged over 5 runs :

**Table 2:** Detailed performance comparison across models and datasets

Method	Dataset	Support	Precision	Recall	F1-score	Inference (ms)	Train (s)
RFC	Data 1	360	0.97	0.95	0.96	12	0.08
MLP	Data 1	360	0.97	0.92	0.94	15	0.08
PointNet	Data 1	360	0.96	0.93	0.94	30	1.2
RFC	Data 2	7159	0.97	0.90	0.93	12	0.09
MLP	Data 2	7159	0.76	0.82	0.79	15	0.10
PointNet	Data 2	7159	0.97	0.89	0.93	30	1.3

[R2.5]Across both datasets, RFC matches or exceeds PointNet’s F1-score while outperforming the MLP by 2–14 points, demonstrating robust accuracy on both small and large scales [R1.3; R2.3]. RFC’s average inference latency is 12 ms/frame 2.5 time faster than PointNet and 1.25 time faster than MLP validating time performance without GPU acceleration. Training times for RFC remain under 0.1 s, versus over a second for PointNet, enabling fast retraining and that can be used for continuous learning in dynamic environments.

## Conclusions

In this article, we have introduced a novel approach for people detection from point cloud data, utilizing a Random Forest classifier optimized for both speed and accuracy. Our methodology directly addresses the challenges of processing complex and sparse data from an innovative multi-point LiDAR system, which significantly enhances the quality and resolution of the data collected. This technological integration facilitates a robust framework for detecting human figures in various environmental conditions, emphasizing the adaptability and efficiency required for real-time processing.

Our extensive experiments validate the effectiveness of our approach, showcasing its superiority over traditional methods like the Multi-Layer Perceptron (MLP) in handling larger datasets. These results highlight our method’s potential for broader application across diverse domains such as autonomous driving, public safety, and interactive robotic systems. The ability to perform accurate and rapid people detection in point clouds opens up significant opportunities for enhancing situational awareness and safety in these areas.

The scalability and versatility of the Random Forest classifier render it an attractive choice for extending our approach to other object recognition tasks within point cloud analysis. With its potential applications to autonomous driving, surveillance, human-robot interaction, industrial environments, safety, and security, our proposed approach holds promise for a safer and more efficient interactions between humans and intelligent systems.

In summary, our proposed approach not only opens new avenues for advancing real-time human tracking in point cloud perception but also contributes to the broader objective of enhancing situational awareness and using intelligent systems to effectively perceive and interact with dynamic environments.

Looking ahead, future research directions may encompass further comparisons with existing and emerging methodologies, exploration of additional sensor modalities for integration, continued optimization of the classification to enhance overall performance, exploration of broader object recognition capabilities, etc.

## Data Availability

The LiDAR data used in this study are not publicly available due to contractual restrictions. However, the data can be provided by the corresponding author upon reasonable request.

## Footnotes

1 Available at: https://segment-anything.com/, accessed in October 2023.

2 Additional details can be found in https://ouster.com/blog/introducing-the-os-1-128-lidar-sensor/ accessed in October 2023.

## Code Availability

All code generated or used during the study are available from the corresponding author by request.

## Materials Availability

The LiDAR sensor is available for other use cases and applications. Interested parties may contact the corresponding author for further information on access and customization options.

## References

[1] Aalerud A, Dybedal J, Subedi D. Reshaping field of view and resolution with segmented reflectors: bridging the gap between rotating and solid-state lidars. Sensors. 2020. 10.3390/s20123388. 10.3390/s20123388 PMC7348914 32549400

[2] Belbachir A, Ortiz AM, Aalerud A, Belbachir AN. From point cloud perception toward people detection. In: Gini G, Nijmeijer H, Filev DP, editors. Proceedings of the 20th international conference on informatics in control, automation and robotics, ICINCO 2023, Rome, Italy, November 13–15, 2023, vol. 1. SCITEPRESS; 2023. p. 520–6. 10.5220/0012258800003543.

[3] Belgiu M, Drăguţ L. Random forest in remote sensing: a review of applications and future directions. ISPRS J Photogramm Remote Sens. 2016;114:24–31.

[4] Cao C, Preda M, Zaharia T. 3D point cloud compression: a survey. In: The 24th international conference on 3D web technology; 2019. p. 1–9.

[5] Cutler DR, Edwards TC Jr, Beard KH, Cutler A, Hess KT, Gibson J, Lawler JJ. Random forests for classification in ecology. Ecology. 2007;88(11):2783–92. 10.1890/07-0539.1. 18051647 10.1890/07-0539.1

[6] Dai D, Chen Z, Bao P, Wang J. A review of 3D object detection for autonomous driving of electric vehicles. World Electric Veh J. 2021. 10.3390/wevj12030139.

[7] Dalal N, Triggs B, Schmid C. Human detection using oriented histograms of flow and appearance. Eur Conf Comput Vis. 2006;3952:428–41. 10.1007/11744047_33.

[8] Huang X, Mei G, Zhang J, Abbas R. A comprehensive survey on point cloud registration. Preprint arXiv:2103.02690; 2021.

[9] Javaheri A, Brites C, Pereira F, Ascenso J. Subjective and objective quality evaluation of 3D point cloud denoising algorithms. In: 2017 IEEE international conference on multimedia & expo workshops (ICMEW). IEEE; 2017. p. 1–6.

[10] Li Y, Bu R, Sun M, Wu W, Di X, Chen B. Pointcnn: convolution on x-transformed points. In: Bengio S, Wallach H, Larochelle H, Grauman K, Cesa-Bianchi N, Garnett R, editors. Advances in neural information processing systems. vol. 31. Curran Associates, Inc.; 2018. https://proceedings.neurips.cc/paper_files/paper/2018/file/f5f8590cd58a54e94377e6ae2eded4d9-Paper.pdf.

[11] Lin CH, Kong C, Lucey S. Learning efficient point cloud generation for dense 3D object reconstruction. In: Proceedings of the AAAI conference on artificial intelligence; 2018. p. 32.

[12] Qi CR, Su H, Mo K, Guibas LJ. Pointnet: deep learning on point sets for 3D classification and segmentation; 2017.

[13] Qi CR, Liu W, Wu C, Su H, Guibas LJ. Frustum pointnets for 3D object detection from RGB-D data; 2017. CoRR arXiv:abs/1711.08488. http://arxiv.org/abs/1711.08488.

[14] Qi CR, Yi L, Su H, Guibas LJ. Pointnet++: deep hierarchical feature learning on point sets in a metric space. In: Guyon I, Luxburg UV, Bengio S, Wallach H, Fergus R, Vishwanathan S, Garnett R, editors. Advances in neural information processing systems. vol. 30. Curran Associates, Inc.; 2017. https://proceedings.neurips.cc/paper_files/paper/2017/file/d8bf84be3800d12f74d8b05e9b89836f-Paper.pdf.

[15] Qi CR, Yi L, Su H, Guibas LJ. Pointnet++: deep hierarchical feature learning on point sets in a metric space. Adv Neural Inform Process Syst. 2017;30:1.

[16] Qi CR, Hao S, Kaichun M, Guibas LJ. Pointnet: deep learning on point sets for 3D classification and segmentation. In: Proceedings of the IEEE conference on computer vision and pattern recognition; 2017. p. 652–60.

[17] Shi S, Wang X, Li H. Pointrcnn: 3D object proposal generation and detection from point cloud. In: IEEE conference on computer vision and pattern recognition, CVPR 2019, Long Beach, CA, USA, June 16–20; 2019. p. 770–9. Computer Vision Foundation/IEEE 2019; http://openaccess.thecvf.com/content_CVPR_2019/html/Shi_PointRCNN_3D_Object_Proposal_Generation_and_Detection_From_Point_Cloud_CVPR_2019_paper.html.

[18] Simon M, Milz S, Amende K, Gross H. Complex-yolo: real-time 3D object detection on point clouds; 2018. CoRR arXiv:abs/1803.06199. http://arxiv.org/abs/1803.06199.

[19] Uy MA, Pham QH, Hua BS, Nguyen T, Yeung SK. Revisiting point cloud classification: a new benchmark dataset and classification model on real-world data. In: Proceedings of the IEEE/CVF international conference on computer vision; 2019. p. 1588–97.

[20] Yang G, Huang X, Hao Z, Liu MY, Belongie S, Hariharan B. Pointflow: 3D point cloud generation with continuous normalizing flows. In: Proceedings of the IEEE/CVF international conference on computer vision; 2019. p. 4541–50.

[21] Yang Z, Sun Y, Liu S, Shen X, Jia J. STD: sparse-to-dense 3D object detector for point cloud; 2019. CoRR arXiv:abs/1907.10471. http://arxiv.org/abs/1907.10471.

